# Delayed minocycline inhibits ischemia-activated matrix metalloproteinases 2 and 9 after experimental stroke

**DOI:** 10.1186/1471-2202-7-56

**Published:** 2006-07-17

**Authors:** Livia S Machado, Anna Kozak, Adviye Ergul, David C Hess, Cesario V Borlongan, Susan C Fagan

**Affiliations:** 1 Program in Clinical and Experimental Therapeutics, Clinical Pharmacy Department, College of Pharmacy, University of Georgia, Augusta, GA, USA; 2 Veteran's Affairs Medical Center, Downtown Division, Augusta, GA, USA; 3 Vascular Biology Center, Medical College of Georgia, Augusta, GA, USA; 4 Department of Neurology, Medical College of Georgia, Augusta, GA, USA

## Abstract

**Background:**

Matrix metalloproteinases 2 and 9 (MMP-2 and MMP-9) are increased in the brain after experimental ischemic stroke in rats. These two proteases are involved with the degradation of the basal lamina and loss of stability of the blood brain barrier that occurs after ischemia and that is associated with thrombolytic therapy in ischemic stroke. Minocycline is a lipophilic tetracycline and is neuroprotective in several models of brain injury. Minocycline inhibits inflammation, apoptosis and extracellular matrix degradation. In this study we investigated whether delayed minocycline inhibits brain MMPs activated by ischemia in a model of temporary occlusion in Wistar rats.

**Results:**

Both MMP-2 and MMP-9 were elevated in the ischemic tissue as compared to the contra-lateral hemisphere after 3 hours occlusion and 21 hours survival (p < 0.0001 for MMP-9). Intraperitoneal minocycline at 45 mg/kg concentration twice a day (first dose immediately after the onset of reperfusion) significantly reduced gelatinolytic activity of ischemia-elevated MMP-2 and MMP-9 (p < 0.0003). Treatment also reduced protein concentration of both enzymes (p < 0.038 for MMP-9 and p < 0.018 for MMP-2). *In vitro *incubation of minocycline in concentrations as low as 0.1 μg/ml with recombinant MMP-2 and MMP-9 impaired enzymatic activity and MMP-9 was more sensitive at lower minocycline concentrations (p < 0.05).

**Conclusion:**

Minocycline inhibits enzymatic activity of gelatin proteases activated by ischemia after experimental stroke and is likely to be selective for MMP-9 at low doses. Minocycline is a potential new therapeutic agent to acute treatment of ischemic stroke.

## Background

Matrix metalloproteases (MMPs) are a family of zinc dependent proteases responsible for the extracellular matrix turnover and degradation of bioactive proteins. In cerebral ischemia, MMPs 2 and 9, also designated as gelatinases A (72 kDa) and B (92 kDa) have been identified to mediate the degradation of the basal lamina [1,2] and hemorrhagic transformation [3]. MMPs 2 and 9 have been shown to be elevated a few hours after ischemia [4] and to maintain increased activity for days after the onset [2,5]. The inhibition of these enzymes by specific class inhibitors reverts breakdown of laminin [6] and prevents increased barrier permeability, edema, and hemorrhage after ischemic stroke [7-9]. The development of MMP inhibitors as therapeutic agents has been limited by their poor solubility.

Minocycline is a commonly used semi-synthetic tetracycline with anti-inflammatory and anti-apoptotic properties [10,11]. Minocycline interferes with MMP activity [12,13] and has been shown to be neuroprotective in cerebral ischemia [14] and in other models of brain injury [15,16].

In this study we investigated whether minocycline prevents ischemia-induced MMP activation in a temporary model of temporary focal cerebral ischemia in rats, and explored the dose-response relationship and possible specificity of *in vitro *MMP-inhibition by this drug. Because MMP-2 and MMP-9 have distinct mechanisms of activation and different roles after stroke, the potential selectivity of minocycline for either enzyme should be determined. Furthermore, MMP inhibition may be the central mechanism through which minocycline provides neurovascular protection [17]. There are currently no drugs marketed specifically for their ability to inhibit systemic MMPs. Minocycline could represent a new clinical approach to inhibiting acute MMP activation, reducing the risk of development of hemorrhage and protecting the patient from further damage after acute ischemic stroke.

## Results

### MMP activity after ischemic stroke

**Figure 1:**
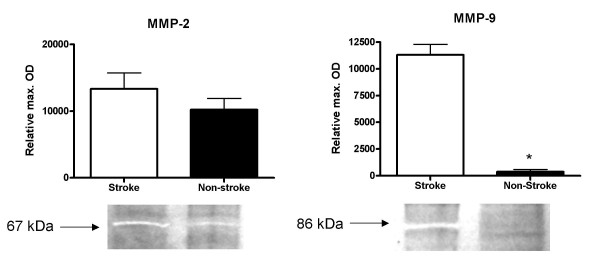
The ischemic hemisphere has increased MMP-9 (significant increase) and MMP-2 activity as compared to the contra-lateral non ischemic control hemisphere in non-treated animals. Sample sizes: n = 8 for both PBS and minocycline treated groups (p < 0.0001 for MMP-9 increase). Each band represents one different replicate sample. Molecular weights were determined with the use of pre-stained protein standards.

**Figure 2:**
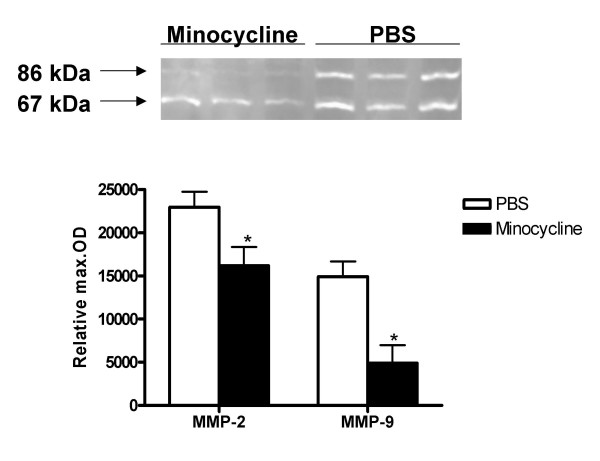
Intra-peritoneal minocycline 45 mg/kg treatment twice a day (first dose immediately after reperfusion), significantly reduced the ischemia-induced increase in MMP-2 and MMP-9 gelatinolytic activities (represented by the active form bands; 67 and 86 kDa respectively). Sample sizes: PBS n = 6; Minocycline n = 8 (p < 0.0003). Each band represents one different replicate sample.

**Figure 3:**
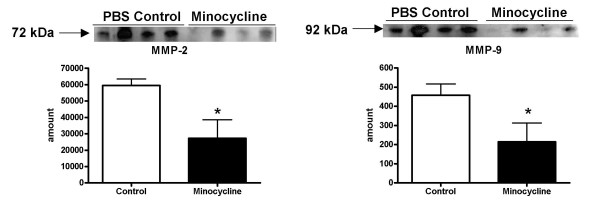
Intra-peritoneal minocycline 45 mg/kg treatment twice a day (first dose immediately after reperfusion), significantly reduced the ischemia-induced increase in MMP-2 and MMP-9 total protein concentrations as compared to PBS treated control animals. Sample sizes: PBS n = 6; Minocycline n = 6 (p < 0.038 for MMP-9 and p < 0.018 for MMP-2). Each band represents one different replicate sample.

The 3 hour ischemia followed by 21 hour survival after reperfusion caused increased activity of MMP-2 and MMP-9 in the ischemic hemisphere (Fig. 1) as compared to the contra-lateral hemisphere (n = 8, p < 0.0001 for MMP-9, MMP-2 activity was increased, but not significantly). The densitometric analysis revealed also that intra-peritoneal minocycline 45 mg/kg treatment twice a day (first dose immediately after reperfusion), significantly reduced the ischemia-induced increments in both MMP-2 and MMP-9 active gelatinolytic forms (n = 6 for control and 8 for treated animals) (p < 0.0003) (Fig. 2). The same treatment regimen with intra-peritoneal minocycline also significantly reduced the ischemia-induced increase in MMP total protein concentration (Fig. 3) (n = 6 for PBS and 8 for minocycline treated groups, p < 0.038 for MMP-9 and p < 0.018 for MMP-2).

### Neurological evaluation

All animals had a significant deficit (scored 3 points in the Bederson Scale) prior to reperfusion, demonstrating successful MCAO. The neurological scores did not show a significant difference between the treatment and control groups. The scale shows a high variability between animals and a much higher sample size is required to detect a significant difference.

### *In vitro *MMP inhibition by minocycline

**Figure 4:**
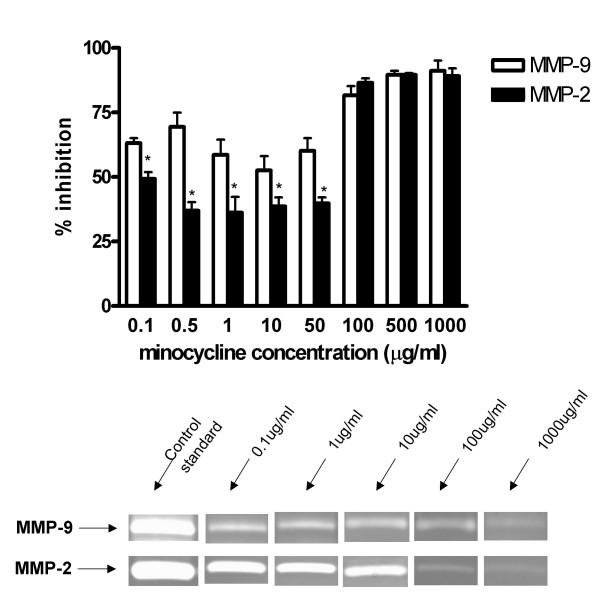
Minocycline concentrations ranging within 0.1 and 1000 μg/ml inhibits the activity of 0.05 ng recombinant human MMP-2 and MMP-9 as compared to the control enzymatic activity. At low concentrations, MMP-9 inhibition is greater than that of MMP-2 (p < 0.05).

The densitometric analysis revealed that minocycline at 0.1 μg/ml, 0.5 μg/ml, 1 μg/ml, 10 μg/ml, 50 μg/ml, 100 μg/ml, 500 μg/ml and 1000 μg/ml concentrations significantly inhibited the activity of recombinant MMP-2 and recombinant MMP-9 as compared to the control enzymatic activity. The inhibitory effect of minocycline in both enzymes was similar or slightly greater than the positive control EDTA in 1 μg/ml concentration (not shown). There was a significant interaction between the two proteases and minocycline drug concentration such that, at concentrations of 100 ug/ml and below, the percent inhibition is significantly higher for MMP-9 (p < 0.05) (Fig 4).

## Discussion

This study demonstrated for the first time that delayed treatment with minocycline inhibits MMPs that are elevated after temporary experimental cerebral ischemia. In addition, this inhibitory effect is present over a wide range of clinically relevant and safe concentrations of minocycline. In fact, the minocycline concentration expected in the brain after a normal dose of 200 mg in humans (3 mg/kg in rodents), is equivalent to 0.5–1 μg/ml [18] and is higher than the lowest *in vitro *doses tested. Furthermore, our results reinforce previous findings that minocycline is a potent inhibitor of MMP-9 *in vivo *[19] and *in vitro *[13] and suggests a sensitivity of this enzyme for the drug when compared to MMP-2. Our drug dosing regimen was based on previous studies demonstrating therapeutic efficacy of intra-peritoneal minocycline 45 mg/kg in experimental stroke [14,18] and to achieve a constant plasma concentration since the timing of MMP activation spike has not yet been identified either in humans or in rodents. Intra-peritoneal delivery of minocycline in our model of stroke also insures that regardless of differences in the pharmacokinetics of this drug in rodents, the drug will be present whenever the activation of the MMP enzymes occurs, as the delivery of the drug is slow and constant.

Minocycline has been shown to be neuroprotective [14,20,21]. One common pathophysiological mechanism of models of brain ischemic damage is the dysregulation of the proteolytic cascade at the level of the endothelial and microglial cells. Interference of this cascade by minocycline might be the central pathway for these neurovascular protective properties of decreasing tissue injury and also providing functional recovery. Ischemic stroke activates a complex cascade of events [22]. The first tissue damage occurs as early as a few minutes after the occlusion. Within hours after stroke onset, endothelial and inflammatory cells respond to ischemia releasing proteases MMP-2 and MMP-9 that culminate in degradation of the basal lamina and ultimate weakening of the blood brain barrier [23]. The subsequent events are leakage of leukocytes into the parenchyma, and brain edema followed by neuronal death that occurs in the later hours of ischemia. Since MMP-2 and MMP-9 are believed to be largely responsible for the degradation of the blood brain barrier and also involved in the signaling of neuronal cell death [24,25], the inhibition of the proteolytic cascade is a logical target for several models of brain injury that involve matrix degradation and vascular instability [26].

Our studies point to an apparent selectivity of minocycline for MMP-9 with low dose minocycline. This could reflect either a time profile difference between the activation of the two enzymes, the pathway minocycline interferes, or a chemical selectivity for MMP-9. Among the main differences between their molecular pathways, MMP-2 is constitutively expressed, while MMP-9 can be stimulated at the level of gene activation by multiple stimuli including ischemia. Both enzymes are expressed as pro-forms and after they are secreted they require chemical or proteolytic activation to become functional [27]. In endothelial brain cells, little is known about the presence and regulation of the different MMPs [28,29]. Further studies are necessary to determine the mechanisms of activation of MMPs in the brain after ischemia and the mechanism by which minocycline decreases MMP activity.

Minocycline has in its chemical structure an active binding site that confers the ability to chelate divalent ions. Our *in vitro *studies confirm that minocycline is able to inhibit gelatin digestion by MMPs by interacting with these enzymes. MMPs require zinc in their active site for functional activity and removal of the zinc results in change of conformation and inactivation of the enzyme. Tetracyclines have been shown to inhibit collagenolysis [30,31] and inhibit MMP-9 activity [32]. Previous studies, however, have shown that multiple tetracyclines can inhibit collagenase MMPs via down-regulation of gene expression in a cellular model of rheumatoid arthritis using cultured chondrocytes [33] and that pre-treatment with minocycline down-regulates pro-MMP expression after permanent cerebral ischemia [17]. Furthermore, because minocycline has been shown to be neuroprotective in different models of brain injury, it is likely to act by multiple mechanisms, with MMP inhibition being a central link for its anti-inflammatory and anti-apoptotic properties in the ischemic cascade and reperfusion. Our studies are pioneer in investigating the ability of minocycline to decrease MMP activity in the setting of a temporary model of stroke, and with treatment occurring post reperfusion. Increase in MMP activity and its consequences are relevant in stroke from the standpoint of ischemic damage itself and also from the standpoint of delayed reperfusion damage alone.

In order to determine the clinical relevance of these findings for acute stroke treatment, the next step is to determine if the MMP inhibition by minocycline in a model of temporary occlusion will result in decreased blood brain barrier degradation and decreased hemorrhage formation. In addition, the vascular protection properties of minocycline should also be studied for ischemic stroke after treatment with tissue plasminogen activator (tPA). tPA is currently the only available acute treatment for ischemic stroke patients but its application is limited due to the increased risk of serious intracerebral bleeding [34]. Because tPA is an extracellular protease and is not highly specific for plasminogen cleavage, reperfusion with tPA might amplify the MMP damage in the brain as has been previously speculated [35,36] and concurrent administration of minocycline with tPA or other reperfusion agents might be a potential approach for reestablishing blood flow without compromising the vasculature. These studies are currently ongoing.

## Conclusion

Our studies provide evidence that the inhibition of MMP enzymatic activity can be achieved with intraperitoneal treatment of minocycline after experimental ischemic stroke. MMP inhibition is likely a mechanism through which minocycline is neuroprotective in stroke. Furthermore, the *in vitro *inhibitory effect of minocycline is observed at very low therapeutic concentrations with MMP-9 being more. Because thrombolytic agents exacerbate the proteolytic cascade, minocycline raises optimism for the acute treatment of ischemic stroke patients. Minocycline is a candidate neuroprotective agent to be used in combination with tPA.

## Methods

### Drug preparation and regimen

Minocycline powder was purchased from Sigma Aldrich CO., Saint Louis, MO. The drug was prepared fresh one hour prior to administration by dissolution in phosphate buffered saline (PBS) pH 7.4 (Fisher Scientific, Pittsburgh, PA). The preparation tube was covered with aluminum foil and protected from light. Minocycline was administered intraperitoneally to animals in two doses of 45 mg/kg, the first one being 5 minutes after the onset of reperfusion and the second one 12 hours later. This dosing regimen was based on previous studies [14,18]. The injection was 9 ml/kg. Animals were randomized between the minocycline treated group and the PBS treated group and received equivalent injection volumes.

### Animal procedures and experimental stroke

The Institutional Animal Care and Use Committee (IACUC) of the Veterans Affairs Medical Center approved the protocol. Male Wistar rats purchased from Charles River Laboratory (Wilmington, MA) were used. Animals used were within a range of 270–300 g of body weight. Cerebral ischemia was induced by intraluminal suture occlusion of the MCA [37]. A 20 mm 3.0 nylon monofilament with round tip was inserted through the right internal carotid artery up to the origin of the MCA. After 3 hours of ischemia the animals were reperfused by removing the suture. The animals were anesthetized with isofluorane inhalation in a glass chamber prior to stroke procedure and reperfusion. The anesthesia was kept by 2% isofluorane during surgery. Body temperature was maintained with heating pad during surgery and reperfusion. The animals were evaluated neurologically and for motor ability using the Bederson Scale [38] prior to reperfusion and sacrifice. Prior to sacrifice, which occurred 24 hours after stroke, the animals were anesthetized with a cocktail of ketamine (45 mg/kg) and xylazine (15 mg/kg) via intramuscular injection. The animals were then perfused with ice cold PBS, sacrificed and the brains were extracted.

### Tissue processing, immunoblotting and gelatin zymography

After extraction, the brain was washed with PBS and placed in a coronal matrix. The olfactory bulb and the first 2 mm-thick slice of the anterior brain were discarded. Four consecutive 2 mm slices corresponding to the major infarcted area and the contra-lateral hemisphere were separated as a block. The ischemic and non ischemic hemispheres were then separated, placed in cryotubes and flash frozen in liquid nitrogen. The tubes were stored in a -80°C freezer until homogenization. The samples were homogenized in 450 μL of cold working buffer containing 50 mM Tris-HCl (pH 7.5), 75 mM NaCl, and 1 mM PMSF as described by Heo and collaborators [3]. The tissue was processed with a homogenizer for 10 minutes and centrifuged at 4°C for 20 minutes at 13000 rpm. The supernatants were separated, frozen and kept at -80°C until use. The total protein concentration was assessed by the Bradford method.

On the day of the experiment, the volume equivalent to 50 μg of total protein was loaded into fresh made 10% polyacrylamide gels (Immunoblotting) or gelatin zymography gels. For the zymography experiments, the gels were electrophoretically separated under non reducing conditions and 100 V. After electrophoresis the zymogram gels were washed in 125 ml 2.5% Triton twice for 20 minutes. The gels were then incubated in activation buffer (Zymogram Development Buffer, Bio-Rad, Hercules, CA) for 20 hours at 37°C. The next day, the gels were stained with Coomassie Blue R-250 Staining Solution (Bio-Rad) for 3 hours and destained for 25 minutes with Destain Solution (Bio-Rad). The gelatinolytic activity of the samples was assessed by densitometric analysis (Gel-Pro v 3.1, Media Cybernetics, Carlsbad, CA) of the bands as a relative comparison to a standard band of recombinant enzyme. To minimize inter-gel variability, all gels had a control lane loaded with 0.5 ng recombinant enzyme, which was used as a standard optical density and enzyme amount (in ng). The lytic bands identified in the zymogram gels were subjected to molecular weight identification with the use of pre-stained standard protein marker (Bio-Rad). For the immunoblotting experiments, the gels were transferred into nitrocellulose membranes for one hour. Membranes were blocked with blotting grade blocker non- fat dry milk (Bio-Rad). After washing with 0.1% tween 20- tris- buffered saline (TTBS), the membranes were incubated with either anti MMP-2 human (mouse) monoclonal antibody (Oncogene Research Products) or anti MMP-9 monoclonal mouse antibody overnight at 4°C. Membranes were washed again in TTBS, incubated with secondary antibody (goat anti-mouse IgG, horseradish peroxidase conjugated antibody, Calbiochem) for one hour and finally developed with horseradish peroxidase development solution (ECL advance detection kit, Amersham). The membranes were exposed to autoradiography films (Hyblot CL, Denville Scientific Inc.). The density of the sample bands for the immunoblots and zymograms were expressed as maximal optical density relative to the standard band.

### *In vitro *MMP inhibition

The ability of minocycline to inhibit MMPs was tested by a direct, cell free *in vitro *system, in which minocycline was allowed to incubate at room temperature with recombinant active MMP-2 and MMP-9 (Oncogene, EMD Biosciences, La Jolla, CA). Each enzyme was incubated separately. 0.05 nanogram of recombinant enzyme (1:10,000 dilution from the 5 μg/50 μl commercial stock) was incubated with a range of clinically relevant concentrations: 0.1, 0.5, 1, 10, 50, 100, 500 and 1000 μg/ml of minocycline prepared in deionized purified water. Two control samples were used: the negative control in which the enzyme was incubated with no drug, and the positive control in which the enzyme was incubated with 10 μg/ml EDTA. The samples were incubated for 2 hours and separated on 10% zymogram gels as described above. The gels were allowed to run in 100 V under non- reducing conditions. After electrophoresis, the gels were washed twice with 2.5 % Triton for 20 minutes each cycle. They were then incubated for 20 hours with Zymogram Development Buffer at 37°C. The gels were stained with Coomassie Blue R-250 (Bio-Rad) for 3 hours and destained with Destain Solution (Bio-Rad) for 25 minutes. The gelatinolytic bands were analyzed with densitometry (GelPro).

### Statistics

A 2 MMP type (MMP-2 and MMP-9) by 2 group, (saline or minocycline), analysis of variance (ANOVA) on the ranks of the enzyme activity was used to determine the differences in MMP activity, treatment group and their interaction. A 2 MMP type (MMP-2 or MMP-9) by 6 concentrations (minocycline 0.1 μg/ml, minocycline 0.5 μg/ml, minocycline 1 μg/ml, minocycline 100 μg/ml, minocycline 500 μg/ml, and minocycline 1000 μg/ml) ANOVA was used to determine differences in percent inhibition for MMP type, minocycline concentration, and their interaction. A Tukey's test was used to adjust for post-hoc multiple comparisons. Statistical significance was determined at p < 0.05 and SAS 9.1.3 was used for all analyses.

## Authors' contributions

LSM assisted with surgeries, prepared and administered the treatments, performed zymography and prepared the manuscript. AK performed the stroke surgeries and contributed to the preparation of the manuscript. AE contributed to the optimization of zymograms, data interpretation and preparation of the manuscript. DCH and CVB contributed to the design, interpretation and preparation of the manuscript. SCF coordinated all aspects of this work and the manuscript. All authors approved the final version of the manuscript.
